# Identification of TLR2/TLR6 signalling lactic acid bacteria for supporting immune regulation

**DOI:** 10.1038/srep34561

**Published:** 2016-10-06

**Authors:** Chengcheng Ren, Qiuxiang Zhang, Bart J. de Haan, Hao Zhang, Marijke M. Faas, Paul de Vos

**Affiliations:** 1 Immunoendocrinology, Division of Medical Biology, Department of Pathology and Medical Biology, University of Groningen and University Medical Center Groningen, Hanzeplein 1, 9700 RB Groningen, The Netherlands; 2 School of Food Science and Technology, Jiangnan University, 1800 Lihu Road, Wuxi 214122, China

## Abstract

Although many lactic acid bacteria (LAB) influence the consumer’s immune status it is not completely understood how this is established. Bacteria-host interactions between bacterial cell-wall components and toll-like receptors (TLRs) have been suggested to play an essential role. Here we investigated the interaction between LABs with reported health effects and TLRs. By using cell-lines expressing single or combination of TLRs, we show that LABs can signal via TLR-dependent and independent pathways. The strains only stimulated and did not inhibit TLRs. We found that several strains such as *L. plantarum* CCFM634, *L. plantarum* CCFM734, *L. fermentum* CCFM381, *L. acidophilus* CCFM137, and *S. thermophilus* CCFM218 stimulated TLR2/TLR6. TLR2/TLR6 is essential in immune regulatory processes and of interest for prevention of diseases. Specificity of the TLR2/TLR6 stimulation was confirmed with blocking antibodies. Immunomodulatory properties of LABs were also studied by assessing IL-10 and IL-6 secretion patterns in bacteria-stimulated THP1-derived macrophages, which confirmed species and strain specific effects of the LABs. With this study we provide novel insight in LAB specific host-microbe interactions. Our data demonstrates that interactions between pattern recognition receptors such as TLRs is species and strain specific and underpins the importance of selecting specific strains for promoting specific health effects.

Lactic acid bacteria (LAB) have been traditionally utilized for manufacturing fermented foods and are associated with a broad variety of beneficial health effects^1^. LAB also belong to the indigenous commensal microflora in humans and animals^2^. Some LAB strains have been confirmed to exert health-promoting effects in both animal studies and human clinical trials and are sometimes used as probiotics^1^. However, LAB strains from different species or even strains within the same species might possess totally different regulatory properties. Moreover, even though some LAB strains have been demonstrated to be beneficial for the remission of some diseases, they are not beneficial under all clinical circumstances^3^, and effects of some LABs are different in diseased and healthy individuals^4^,^5^. Therefore, it is imperative to select specific LAB strains for prevention or cure of specific disorders^6^.

Many LABs exhibit *in vivo* immunomodulatory properties by supporting intestinal immune cell responses^7^, reinforcing intestinal barrier function^8^, and inhibiting pathogenic adhesion^9^. Crosstalk between LAB and host cells in the gastrointestinal mucosa is a key determinant for modulatory activities^10^,^11^. Furthermore, recognition of LAB by the intestinal immune system is crucial for transducing their regulatory benefits to the host, which is initiated via the ligation of microbe-associated molecular patterns (MAMPs) exhibited on LAB to pattern recognition receptors (PRRs) expressed on immune cells and epithelial cells^12^. Toll-like receptors (TLRs) are the most important and best-characterized class of PRRs. At least nine human TLRs have been studied and recognize specific MAMPs on different microorganisms^13^.

TLRs play a cardinal role in recognizing microbial components by the innate immune system, which leads to downstream signalling cascades, inducing an immune response. Following binding of ligands to TLRs, adaptor molecules such as myeloid differentiation primary-response protein 88 (MyD88) will be recruited for transmitting downstream signalling responses. MyD88-dependent pathways comprise the vast majority of TLRs signalling including TLR2, 4, 5, 7, 8, and 9 although MyD88-independent pathways are indispensable for TLR3 signalling^13^. In relation to LABs, TLR2 and MAMPs have been studied in most detail. MAMPs on LAB cell-surfaces are peptidoglycan, lipoteichoic acid, and wall teichoic acid which are predominantly detected by TLR2^12^. So far, a variety of studies have demonstrated that TLR2 signalling is a pivotal player in maintaining immune homeostasis especially in the intestine, which includes promoting repair of damaged epithelial mucosa, inhibiting intestinal inflammation and enhancing intestinal barrier integrity^14^,^15^,^16^,^17^. Dysregulation of TLR2 signalling is widely believed to be associated with a range of diseases such as autoimmune diseases and chronic inflammation^18^,^19^. This dual role is caused by the unique property of TLR2 signalling pathways that it can only be activated in the presence of TLR1 and TLR6^20^,^21^. TLR2/TLR1 activation was shown to induce pro-inflammatory cytokines such as IL-17 and IL-12 while TLR2/TLR6 activation could elicit tolerogenic IL-10 secretion^22^,^23^,^24^.

It is worth noting that even though MAMPs on LAB share similar conserved basic structure, subtle structural distinctions of MAMPs among various LAB species and strains can result in activation of different TLRs pathways, thus resulting in differential modulatory properties^25^,^26^,^27^. We therefore screen in this study for all nine TLRs. Also costimulation of TLR2 and TLR6 was studied since TLR2 in combination with TLR6 triggers beneficial regulatory immune responses as described above. It is assumed that LAB strains stimulating TLR2/TLR6 pathways might have promising potentials for modulating excessive inflammatory reactions such as may occur in inflammatory bowel disease^22^. To identify whether LABs are capable of triggering TLR2/TLR6 signalling pathways, 16 different LAB strains of food and human origins were characterized and tested for their interactions with specific TLRs. The selection of these strains was based on a literature research for strains or parental strains that have reported health benefits^28^,^29^,^30^,^31^,^32^,^33^,^34^,^35^.

## Results

### Modulation of pro-and anti-inflammatory cytokines production in THP1 macrophages by different bacterial strains

**Figure 1:**
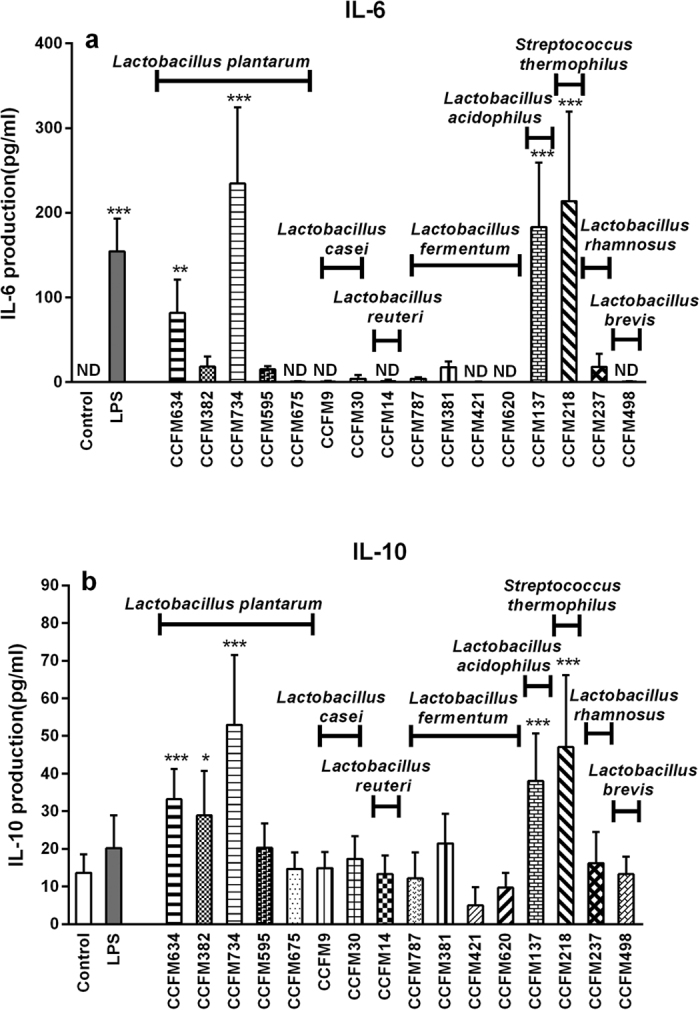
LAB-induced cytokine production in PMA-stimulated THP1 macrophages. PMA-stimulated THP1 macrophages were treated with different bacteria (bacteria to cells ratios of 40:1) or 1 μg/ml LPS (positive control) for 24 h. Assays were performed in duplicate wells. Untreated THP1 macrophages served as negative control. IL-6 and IL-10 levels in the supernatant were measured by ELISA. The results shown represent mean and standard deviation (SD) of four independent experiments. ND, not detectable. Statistical significance between different treatment groups and untreated control group was measured using one-way ANOVA with application of the Bonferroni multiple comparisons test (*p < 0.05; **p < 0.01, ***p < 0.001).

A large number of species that potentially might serve as immune-active bacterial strains were selected from the Culture Collections of Food Microbiology^28^,^29^,^30^,^31^,^32^,^33^,^34^,^35^. In order to assess the immune-modulatory capacity of the various bacterial strains, IL-6 and IL-10 secretion in LAB-stimulated PMA-differentiated THP1 macrophages were studied. As shown in Fig. 1, different bacterial species triggered differential cytokines production profiles. Generally, *Lactobacillus (L.) plantarum*, *L. acidophilus* and *Streptococcus (S.) thermophilus* possessed stronger activation potency of both IL-6 and IL-10 production than other species such as *L. fermentum, L. casei*, *L. reuteri*, and *L. brevis*. Moreover, strains within the same species such as *L. plantarum* also could differentially induce cytokines secretion in immune cells. *L. plantarum* CCFM634 and *L. plantarum* CCFM734 initiated higher cytokines production when compared with *L. plantarum* CCFM 382 *L. plantarum* CCFM595, and *L. plantarum* CCFM675.

LPS, a generally accepted pro-inflammatory agent, could barely induce IL-10-production in THP1 macrophages, whereas it could induce a profound IL-6 response. On the contrary, LAB strains such as *L. plantarum* CCFM634, *L. plantarum* CCFM734, *L. acidophilus* CCFM137 and *S. thermophilus* CCFM218 could dominantly trigger both IL-6 and IL-10 secretion (*p* < 0.01, *p* < 0.001). Besides, it’s worth noting that IL-10 production induced by the latter three strains were much higher than that induced by LPS.

The IL-10 responses here were in the concentration range from 0 to 80 pg/ml. It might be suggested that these are low when compared to other cell sources such as murine bone marrow derived dendritic cells (BMDCs)^25^. However, human macrophage-like THP1 cells^36^,^37^,^38^,^39^ but also human primary dendritic cells such as umbilical cord blood-derived DCs^10^,^11^ have a IL-10 response in this range^10^,^11^,^36^,^37^,^38^,^39^.

### NF–κB/AP-1 activation induced by different bacterial strains was strain and dose-dependent

**Figure 2:**
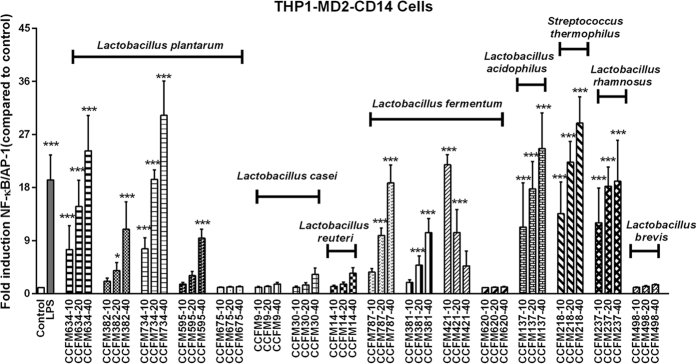
Bacteria-induced differential activation of NF-κB/AP-1 in THP1-XBlue^TM^-MD2-CD14 cells species- and dose-dependently. SEAP production in cell culture supernatants was measured to assess NF-κB/AP-1 activity as described in Materials and Methods section. For each strain, −10, −20 and −40 represent different bacteria/cells ratios respectively. NF-κB/AP-1 activity is presented as percentage of untreated control cells. The results shown represent mean and standard deviation (SD) of three independent experiments. Statistical significance between different treatment groups and untreated control group was measured using one-way ANOVA with Bonferroni multiple comparisons test (*p < 0.05; **p < 0.01, ***p < 0.001).

Next it was studied whether the species and strain specific effects on cytokine production can be explained by differential effects on pattern recognition receptors (PRRs) activation. To this end, NF-κB/AP-1 activation induced by bacteria in THP1-XBlue^TM^-MD2-CD14 reporter cells was measured. This cell line expresses all TLRs^40^. As shown in Fig. 2, different species of bacteria possessed differential ability to activate NF-κB/AP-1 responses. *L. plantarum*, *L. fermentum*, *L. acidophilus*, *S. thermophilus*, and *L. rhamnosus* triggered the highest NF-κB/AP-1 activation when compared with species such as *L. casei*, *L. reuteri*, and *L. brevis*. Moreover, different strains within the same species could induce NF-κB/AP-1 responses to different degrees. A representative example is the *L. plantarum* species: among this species, *L. plantarum* CCFM634 and *L. plantarum* CCFM734 activated NF-κB/AP-1 responses, which were statistically significantly higher than the signals induced by *L. plantarum* CCFM595 and *L. plantarum* CCFM382 (*p* < 0.001). Some strains such as *L. plantarum* CCFM675 could barely induce NF-κB/AP-1 activation.

Furthermore, the NF-κB/AP-1 induction by the bacteria tested was dose-dependent. It seemed that for most of the strains tested, SEAP secretion was enhanced as the bacteria concentration (bacteria/cells ratios) increased except for *L. fermentum* CCFM421, which triggered less SEAP production when higher concentration of bacteria were applied.

### MyD88-dependency of bacteria-triggered NF-κB signalling

**Figure 3:**
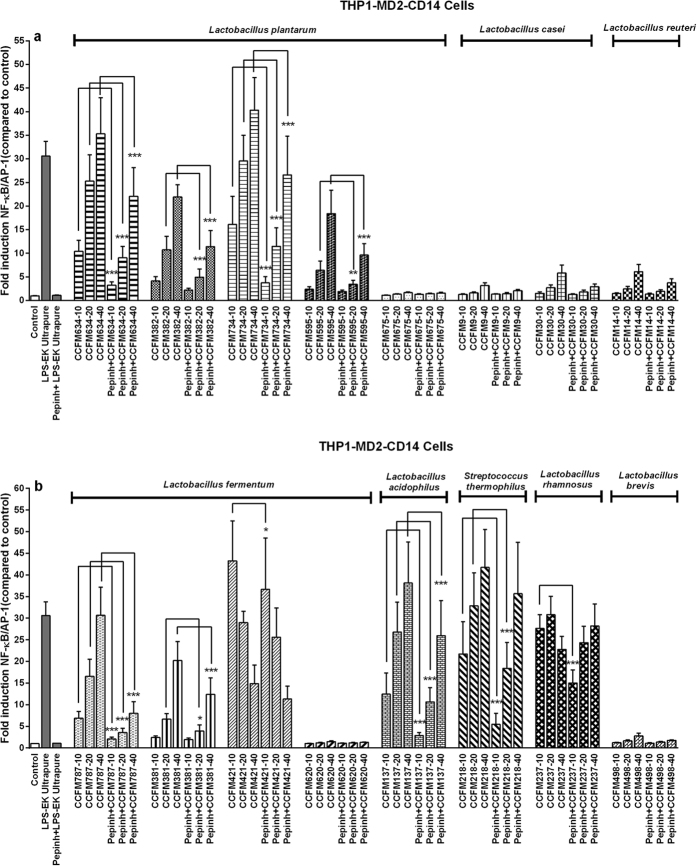
Bacteria signalled via MyD88-dependent pathways. THP1-XBlue^TM^-MD2-CD14 cells were pre-treated with or without MyD88 inhibitory peptide (Pepinh-MYD) for 6 h before stimulation with bacteria. After 24 h of stimulation with bacteria, NF-κB/AP-1 activity was determined. NF-κB/AP-1 activity is presented as percentage of un-stimulated control cells. For each strain, −10, −20 and −40 represent different bacteria/cells ratios respectively. The results shown represent mean and standard deviation (SD) of three independent experiments. Statistical significance between inhibitor-treated groups and respective untreated group was measured using two-way ANOVA test with Bonferroni multiple comparisons test (*p < 0.05; **p < 0.01, ***p < 0.001).

Next, we investigated whether the activation was TLR-dependent by testing the effect of the bacteria on THP-1 reporter cell line pretreated with MyD88 inhibitory peptide Pepinh-MYD. MyD88 is an essential adapter molecule for TLR2, 4, 5, 7, 8, and 9 signalling^13^. Pretreatment with Pepinh-MYD induced a strong and statistically significant reduction in NF-κB/AP-1 responses of most species tested in case of *L. plantarum* (CCFM634, CCFM382, CCFM734, CCFM595), *L. fermentum* (CCFM787, CCFM381, CCFM421), *L. acidophilus* (CCFM137), *S. thermophilus* (CCFM218), and *L. rhamnosus* (CCFM237) (Fig. 3a,b). Moreover, MyD88 inhibitor pretreatment seemed to result in the most marked decrease for CCFM787-initiated activation (more than 70% decrease at the two higher concentrations, *p* < 0.001), which indicated that in this strain MyD88-dependent pathways predominantly mediated the activation of NF-κB/AP-1. The strains such as *L. casei* CCFM30, *L. reuteri* CCFM14, and *L. brevis* CCFM498 were not so much inhibited by the MyD88 inhibitor and therefore predominantly activate NF-κB/AP-1 via Myd88-independent pathways. From the above results, we conclude that the selected bacteria might signal through both MyD88-dependent and MyD88-independent PRR signalling pathways, leading to NF-κB/AP-1 responses.

### Bacteria robustly activated TLR2 signalling pathways in a dose-dependent manner

**Figure 4:**
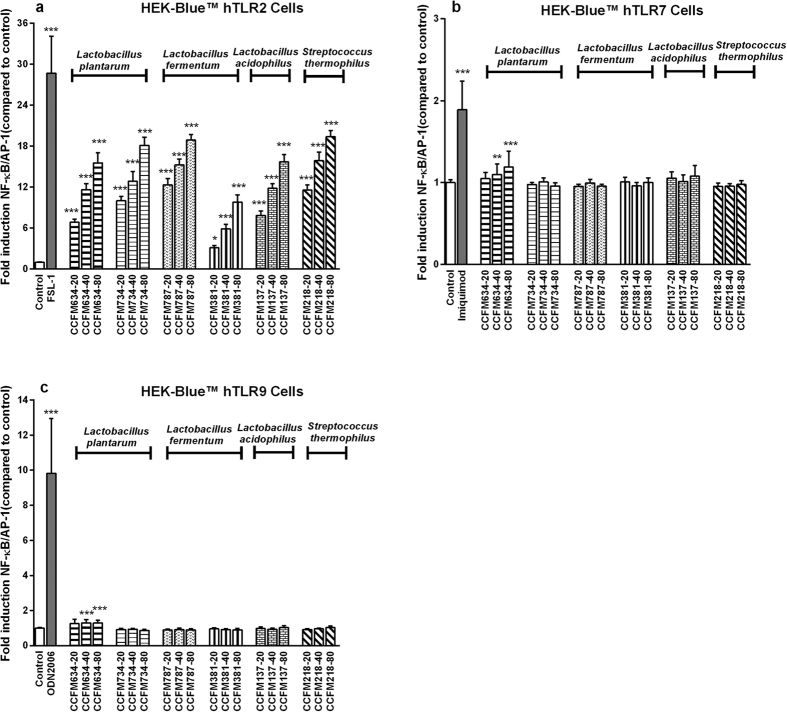
Different bacterial strains strongly activated TLR2 pathways in HEK-Blue^TM^ reporter cells. A series of HEK-Blue^TM^ TLR reporter cell lines were stimulated with various concentrations of strains. After 24 h of co-incubation, SEAP activity in cell culture supernatants was assessed. Respective agonists for TLR served as positive control group. NF-κB/AP-1 activity is presented as percentage of untreated control cells. For each strain, −20, −40 and −80 represent different bacteria/cells ratios respectively. The results shown represent mean and standard deviation (SD) of three independent experiments. Statistical significance between different treatment groups and untreated control group was measured using one-way ANOVA with Bonferroni multiple comparisons test (*p < 0.05; **p < 0.01, ***p < 0.001).

The bacteria that stimulated via TLR-dependent pathways were selected for the next stage in which the specific signalling TLR was identified. To this end HEK reporter cell lines, each carrying one construct for a specific TLR, were stimulated with six selected strains (*L. plantarum* CCFM634, *L. plantarum* CCFM734, *L. fermentum* CCFM787, *L. fermentum* CCFM381, *L. acidophilus* CCFM137, and *S. thermophilus* CCFM218). As shown in Fig. 4, the bacterial strains strikingly activated HEK-Blue™ hTLR2 cells (*p* < 0.05, *p* < 0.001), and slight activation of TLR7 as well as TLR9 was observed by *L. plantarum* CCFM634. Furthermore, the response of bacteria to HEK-Blue™ hTLR2 cells was dose-dependent, which was in accordance with their activation pattern of NF-κB/AP-1 signalling in THP1-XBlue^TM^ reporter cells.

### Bacteria had no dominant inhibitory effect on agonists-induced NF-κB activation

**Figure 5:**
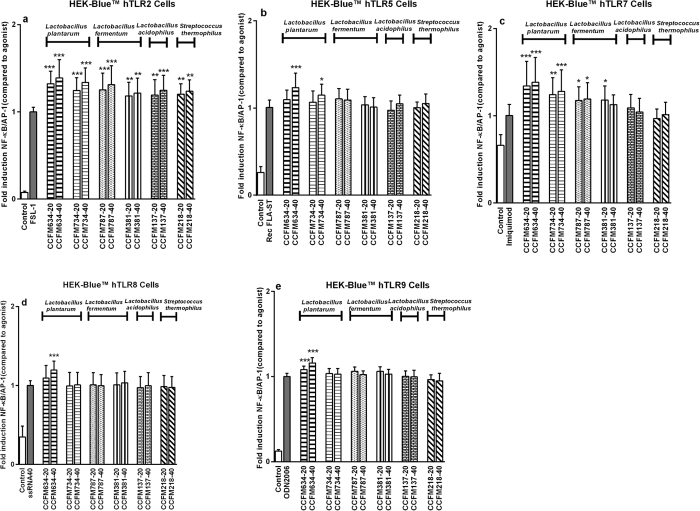
Effect of bacteria on agonists-induced NF-κB/AP-1 activation. HEK-Blue^TM^ TLR reporter cells were stimulated with their agonists and bacteria for 24 h. Then SEAP activity in cell culture supernatants was measured. NF-κB/AP-1 activity is presented as percentage of signals induced by respective agonists. For each strain, −20 and −40 represent different bacteria/cells ratios respectively. The results shown represent mean and standard deviation (SD) of three independent experiments. Statistical significance between different bacteria treatment groups and agonists treatment group was measured using one-way ANOVA with Bonferroni multiple comparisons test (*p < 0.05; **p < 0.01, ***p < 0.001).

Even though activation of TLR signalling is critical for maintaining health, excessive initiation of TLR signalling will be harmful, which might aggregate several diseases^41^,^42^. To evaluate the TLR-inhibitory properties of bacteria, we tested the effect of bacteria on agonists-induced TLR activation in HEK-Blue™ reporter cell lines. However, no remarkable inhibitory effects of agonists-induced TLR activation by various bacteria were found in the HEK-Blue™ cell lines (Fig. 5, Supplemental Fig. S2). Nevertheless, enhancement of a few pre-existed TLR responses were seen with several strains. All the strains tested could augment TLR2 signalling induced by agonists, which is consistent with their predominant activation of TLR2 when HEK-Blue™ hTLR2 cells were only treated with bacteria (Figs 4a and 5a). Additionally, *L. plantarum* CCFM634 elevated agonists-stimulated NF-κB responses in HEK-Blue™ hTLR7,9 cells (Fig. 5c,e), which correlates with its mild activation of TLR7 and TLR9 signalling obtained by co-incubating HEK-Blue™ hTLR7,9 cells with solely bacteria (Fig. 4b,c). Besides, it is worth noting that some other strains tested (*L. plantarum* CCFM734, *L. fermentum* CCFM787, and *L. fermentum* CCFM381) slightly increased TLR7 signalling when compared with agonist-initiated responses, while when only bacteria were added to HEK-Blue™ hTLR7 cells no NF-κB/AP-1 activity was observed (Figs 4b and 5c). This might indicate there’s a synergistic effect between those bacteria and TLR7 agonist. The similar mild up-regulation of NF-κB/AP-1 transcription was also observed in HEK-Blue™ hTLR5, 8 cells (Fig. 5b,d).

### Bacteria were predominantly recognized via TLR2/TLR6 heterodimer

**Figure 6:**
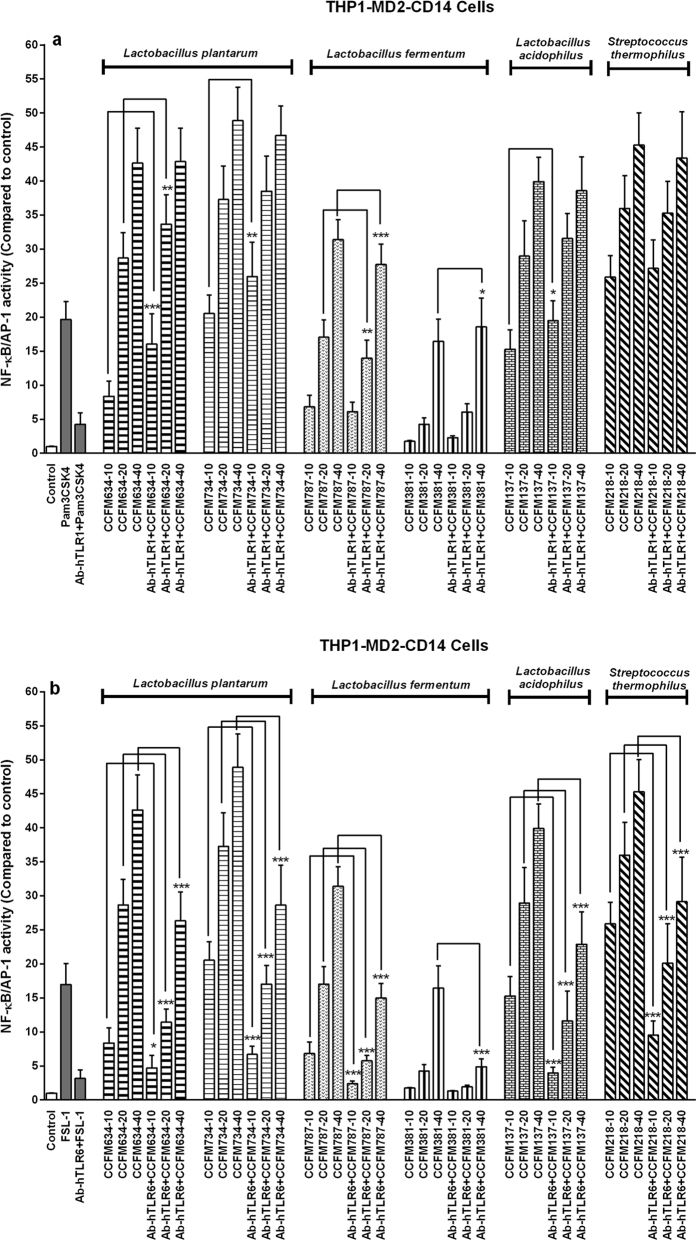
Antibody for TLR1 or TLR6 neutralization assay in THP1-XBlue^TM^-MD2-CD14 cells. THP1-XBlue^TM^-MD2-CD14 cells were pre-treated with or without TLR1 (**a**) or TLR6 (**b**) antibody (Ab-hTLR1 or hTLR6; 0.2 mg/ml) for 1 h, then bacteria was added to cells. After 24 h of stimulation, NF-κB/AP-1 activity was determined. NF-κB/AP-1 activation is presented as percentage of un-stimulated control cells. For each strain, −10, −20 and −40 represent different bacteria/cells ratios respectively. The results shown represent mean and standard deviation (SD) of three independent experiments. Statistical significance between antibody-treated groups and respective untreated group was measured using two-way ANOVA test with Bonferroni multiple comparisons test (*p < 0.05; **p < 0.01, ***p < 0.001).

For full activation, TLR2 must form heterodimers with TLR1 or TLR6^18^. As such, in the HEK293 TLR2 reporter cell line, which also constitutively expresses TLR1 and TLR6, TLR2 dimerizes with TLR1 or TLR6 and activates different pathways^20^,^21^. Several previous studies have validated that TLR2/TLR6 clusters could promote regulatory T (Treg) cells responses, whereas TLR2/TLR1 facilitated pro-inflammatory immune reactions *in vivo*^22^,^23^,^24^. These earlier findings raise a question as to whether TLR1 or TLR6 was involved in the interaction between TLR2 and bacteria in this study. Therefore, TLR1 or TLR6 neutralizing antibody was used separately with THP1-XBlue^TM^-MD2-CD14 cells to block TLR1 or TLR6 signalling. Intriguingly, TLR6 neutralization significantly dampened all the tested strain-induced NF-κB/AP-1 activity (Fig. 6b). On the contrary, TLR1 neutralization appeared to have no effect or even enhanced the signals initiated by some strains (*L. plantarum* CCFM634, *L. plantarum* CCFM734, *L. fermentum* CCFM381, *L. acidophilus* CCFM137, and *S. thermophilus* CCFM218) especially in the two lower concentrations (Fig. 6a). But for the strain *L. fermentum* CCFM787, its activation of NF-κB/AP-1 at three various concentrations were reduced to a lesser magnitude by anti-TLR1 antibody treatment as compared with the effect of anti-TLR6 antibody (Fig. 6a,b). It suggests that both TLR1 and TLR6 were involved in signalling responses induced by *L. fermentum* CCFM787, in which TLR6 prominently mediated its recognition by host immune system. In conclusion, the above observations indicate that TLR2/TLR6 heterodimer were primarily involved in the interactions between the strains tested and host immune cells.

## Discussion

In order to gain insight in the signalling pathways of the LAB strains, we applied a strategic approach. First, THP1 reporter cells expressing all the TLRs were applied for the first screening of LAB strains with PRRs-activating capacities. To this end, 16 LAB strains were selected based on a literature research for strains or parental strains with reported health-promoting functions^28^,^29^,^30^,^31^,^32^,^33^,^34^,^35^. Profound variations among different species and strains of LAB were found. Next MyD88 dependency was confirmed to demonstrate TLRs involvement. MyD88 inhibitor pretreatment caused a strong decrease in activation but not all strains were influenced illustrating that also other, MyD88-independent PRRs, can be involved in beneficial effects of a number of LAB strains. Next we determined in HEK reporter cell lines the specific TLRs involved in the signalling pathways of selected LAB strains. By having insight in the specific TLR-signalling of LAB we can predict for which application they may be used as will be discussed in the next paragraphs.

Many LAB strains have been reported to possess health-promoting functions in consumers, and are used as food or pharmaceutical probiotic formulations^1^. Despite their application it is still largely unknown how specific LAB strains contribute to health. This study was undertaken to provide more insight in how LAB with confirmed health benefits^28^,^29^,^30^,^31^,^32^,^33^,^34^,^35^,^43^,^44^ might contribute to the beneficial effects. Modulation of the immune system has been extensively proposed as an action of some LABs^1^,^6^,^9^. This can occur via effects on the microbiota^6^,^9^, but also by direct contact with immune cells in the small intestine^6^. This direct contact is considered to be specific for the small intestine because of differences in mucus composition. The small intestine has a single, loose and permeable mucus layer, which is distinct from the colon^45^. The colon has both a firm inner and an outer loose mucus layer^45^. The loose mucus structure in the small intestine enables direct interactions between LAB and intestinal cells such as epithelium, macrophages, and dendritic cells as previously reported^12^,^46^,^47^. Ligation of MAMPs to PRRs in LAB-host crosstalk is proposed to determine the immunomodulating capacity of LAB^12^. TLRs have been widely studied as one of the receptors for MAMPS on LAB surfaces^12^,^16^,^17^,^18^. However, as shown here not all LAB strains act via TLRs and many LAB strains may also stimulate other PRRs. This demonstrates that many LAB strains might have TLR independent health benefits. Moreover, our data suggest that every single tested LAB has a unique PRR signalling profile which might be explained by expression of different ligands on the surface of the bacteria or secretion of PRR-stimulating bactericides or other immune active bacterial products^48^,^49^,^50^,^51^,^52^.

In previous studies we have been researching ligands on probiotic strains responsible for immune modulating effect and identified a few potential new MAMPs for immune stimulation^53^,^54^. We also showed that the beneficial immune effects of many bacteria were exerted on the host by supporting Treg cell development^4^,^5^. The generation of Treg cells is highly TLR2/TLR6 dependent and therefore our current experiments were designed to select LABs with Treg supporting effects, i.e. LABs with a TLR2/TLR6 signalling depending effect. As shown in the current study only six of the original 16 strains selected for their health effects^28^,^29^,^30^,^31^,^32^,^33^,^34^,^35^ act via TLR2/TLR6. This underlines the conclusions of our previous studies demonstrating that selection of specific probiotic strains for enforcing specific desired immune responses may be a mandatory strategy for targeted improvement of host health. The six bacteria are of interest as beneficial effects of TLR2-6 signalling have been included in the prevention or management of many diseases such as chronic inflammatory bowel disease and mucositis^22^,^55^.

In all cases we confirmed the involvement of TLR2/TLR6 by application of antibody blocking, in which neutralization of TLR6 led to attenuation of NF-κB activation of all the six LAB strains in THP1 reporter cell line. Unexpectedly, NF-κB signalling initiated by these strains, except for *L. fermentum* CCFM787, appeared to be enhanced by blockade of TLR1. The promoting effects of TLR1 neutralization on bacteria-activated NF-κB/AP-1 responses could be explained as follows. TLR2/TLR1 and TLR2/TLR6 heterodimers are freely spread on the cell surface^56^. TLR1 antibody administration can induce aggregation of TLR2/TLR1 clusters, which would enlarge the surface area on the cell membrane, so that the opportunity of contact and binding between TLR2/TLR6 and microbial ligands would be increased. This would facilitate downstream NF-κB/AP-1 transcription as previously also reported for PRR activation by particulate ß-glucan^56^,^57^.

Intriguingly, in this study we observed that *L. fermentum* CCFM787 had both TLR2/TLR1 and TLR2/TLR6 signalling capacity since not only TLR6 blockade, but also TLR1 blockade resulted in a (modest) diminished NF-κB/AP-1 activity. This suggests a possible bidirectional immunomodulatory role of this strain, of which anti-inflammatory properties seem to be the dominant action as TLR2/TLR6 was more strongly stimulated. However, since TLR2/TLR1 contributes to proinflammatory responses it is not recommended to use this strain in diseases with ongoing inflammation. This finding demonstrates to our opinion the usefulness of our technology platform for selecting specific strains for TLR management and excluding strains from which undesired effects can be predicted.

To date, most studies have been focusing on the PRR activation profile of bacteria^22^,^46^,^58^,^59^,^60^, and not on their inhibitory potentials. Inhibition of TLRs signalling may be of clinical or consumer benefit as in some conditions stimulation of TLRs such as TLR4 may lead to enhanced or sustained inflammation^61^,^62^. To the best of our knowledge we are the first to study inhibition of TLRs by bacteria, but to our surprise, no significant inhibitory effects of the tested LAB strains were observed in this study. What we did observe, however, was synergism in activation between TLR7 agonist and several LAB strains such as *L. plantarum* CCFM734, *L. fermentum* CCFM787, and *L. fermentum* CCFM381. This reveals the existence of synergistic effects of specific LAB strains and ligands of PRRs such as TLR7 agonists which might be an additional mechanism by which bacteria influence health of consumers.

To the best of our knowledge, this is the first study applying a targeted strategy to select LAB strains with specific TLR-dependent signalling capacity. Our screening starts with identifying the possible underlying molecular mechanisms associated with immunomodulatory functions of LAB rather than directly testing in animal- or human-disorders. We feel this is cost- and time-effective as well as selective as we have the opportunity to select the most suitable LAB in a high-throughput fashion.

## Material and Methods

### Preparation of bacteria

All bacterial strains used in this study were provided by Culture Collections of Food Microbiology (CCFM), and listed in Table 1. All strains were cultured in De Man-Rogosa-Sharpe (MRS) broth (Merck, Darmstadt, Germany) under aerobic conditions at 37 °C until stationary phase. Then bacteria were washed twice in phosphate buffered saline (PBS, pH 7.4), suspended at appropriate concentrations in PBS containing 20% glycerol, and stored at −80 °C until used. After freezing viable bacterial counts (CFUs) were quantified by plating serial dilutions on MRS agar.

### Cell cultures

Human monocytic THP1 cell line was obtained from American Type Culture Collection (ATCC), and was cultured in RPMI 1640 medium (Lonza, Verviers, belgium) with 10% fetal bovine serum (Sigma-Aldrich, St. Louis, MO USA), 2 mM L-glutamine (Lonza, Verviers, belgium), 1 mM sodium pyruvate (Lonza, Verviers, belgium), 0.05 mM 2-mercaptoethanol (Scharlau, Barcelona, Spain), 60 μg/ml gentamicin sulfate (Lonza, Verviers, belgium), 2.2 μg/ml amphotericin B solubilized (Sigma-Aldrich, St. Louis, MO USA). THP1-XBlue^TM^-MD2-CD14 and HEK-Blue™ TLR reporter cell lines were purchased from InvivoGen (InvivoGen, Toulouse, France) or home made^63^,^64^. THP1-XBlue^TM^-MD2-CD14 cells are derived from human monocytic THP-1 cell line, and stably express MD2 and CD14 to enhance signalling responses. Each of seven HEK293-Blue™ TLR cell lines was stably transfected with a specific human TLR gene (hTLR2, hTLR3, hTLR4, hTLR5, hTLR7, hTLR8, hTLR9). Both THP1-XBlue^TM^ and HEK-Blue™ cell lines carry NF-κB- and AP-1- inducible secreted embryonic alkaline phosphatase (SEAP) reporter gene. Once TLRs signalling is initiated, NF-κB and AP-1 will be activated, which will initiate the secretion of SEAP. Thus, SEAP activity in the cell supernatants can be used to quantify NF-κB activation. THP1-XBlue^TM^ and HEK-Blue^TM^ cells were cultured as previously described^64^.

### THP1 monocytes to macrophage differentiations and stimulations

THP1 monocytes (1 × 10^6^ cells/ml) were differentiated with 100 ng/ml Phorbol 12-myristate 13-acetate (PMA, Sigma-Aldrich) in T75 cell culture flask (Corning, New York, USA) for 48 h. Then PMA-differentiated THP1 macrophages were detached and resuspended in fresh culture medium at 1 × 10^6^ cells/ml. 0.5 ml of cell suspension were seeded per well in 24-well plates (Corning, New York, USA) and cultured for another 24 h. Subsequently, culture medium were discarded and fresh medium containing various bacterial strains (bacteria to cells ratios of 40:1) or 1 μg/ml LPS (positive control; Invivogen) were added to cells. Un-stimulated cells served as negative control. After 24 h of stimulation, pro- and anti-inflammatory cytokines (IL-6 and IL-10) secretion in the supernatant were measured by ELISA (eBioscience, San Diego, USA) according to manufacturer’s protocol.

### Pattern recognition receptor activation by bacteria in reporter cells

Activation of PRR signalling by various bacterial strains was assessed in THP1-XBlue^TM^ and HEK-Blue^TM^ cells following the manufacturer’s protocol. THP1 and HEK cells were collected and resuspended in fresh culture medium at appropriate cell density (Table 2). Then 100 μl of cell suspension per well was seeded in flat-bottom 96-well plates. For each bacterial strain, initial bacterial suspension stocks were diluted to various concentrations. Cells were stimulated with 10 μl of diluted bacteria suspensions (bacteria to cells ratios of 10:1, 20:1, 40:1), respective agonists (positive control; Invivogen) or PBS containing 20% glycerol (negative control) for 24 h. Then secretion of SEAP in cell supernatants was measured by QUANTI-Blue™ reagent (InvivoGen, Toulouse, France). The types and concentrations of agonists used were listed in Table 2.

### Effect of bacteria on agonists-induced NF-κB activation in HEK-Blue™ cell lines

To investigate the modulatory effect of bacteria on agonists-triggered activation of specific TLR signalling, HEK-Blue™ reporter cells were co-incubated with the corresponding agonist together with different bacteria. HEK-Blue™ reporter cells were seeded at adequate concentrations as indicated in Table 2, using 100 μl of cell suspension for each well. Then appropriate agonists were added to cells, followed by adding diluted different concentrations of bacterial suspensions (bacteria to cells ratios of 10:1, 20:1, 40:1) or PBS containing 20% glycerol (served as negative control for bacteria stimulation group). After 24 h of co-stimulation, SEAP activity in supernatants was determined using QUANTI-Blue™ reagent (InvivoGen, Toulouse, France) according to the manufacturer’s instructions. The concentrations of agonists used were indicated in Table 2.

### MyD88 inhibition assay

MyD88 Inhibitory Peptide Pepinh-MYD (InvivoGen) was used to study whether NF-κB activation by bacteria was MyD88-dependent. THP1-XBlue^TM^ cells were pre-treated with 5 μl of MyD88 inhibitor (1 mM) for 6 h before stimulation with bacteria. After 24 h of stimulation with bacteria (bacteria to cells ratios of 10:1, 20:1, 40:1), SEAP activity in cell culture supernatants was quantified as described above for PRR activation assay.

### Antibody neutralization assay

The role of specific TLR (TLR1 and TLR6) for bacteria-initiated NF-κB signalling were proven by using polyclonal antibodies specific for human TLR1 and TLR6 (InvivoGen). After seeding THP1-XBlue^TM^ cells in plates, 10 μl of specific anti-hTLR1 or anti-hTLR6 (0.2 mg/ml) was added to cells and pre-incubated for 1 h to inhibit the activity of specific TLR. Then cells were co-incubated with bacteria or respective agonists for specific TLR for 24 h. Other procedures were processed the same as in PRR activation assay.

### Statistical analysis

Parametric distribution of data points was confirmed using the Kolmogorov-Smirnov test. Statistical comparisons were performed using one-way ANOVA and two-way ANOVA with Bonferroni multiple comparisons test for post-hoc comparison. GraphPad Prism version 6.0 (San Diego, CA, USA) was used to perform statistical tests. Values of p < 0.05 were considered to be statistically significant. Data are presented as mean ± SD. Significant differences were indicated by asterisks: *p < 0.05; **p < 0.01; ***p < 0.001.

## Additional Information

**How to cite this article**: Ren, C. *et al*. Identification of TLR2/TLR6 signalling lactic acid bacteria for supporting immune regulation. *Sci. Rep.* **6**, 34561; doi: 10.1038/srep34561 (2016).

## Supplementary Material

Supplementary Information

## Figures and Tables

**Table 1:** Bacterial strains used in this study.

Bacterial species	Strain designation	Source or reference
*Lactobacillus plantarum*	CCFM634	CGMCC9740; Chinese Sichuan pickle isolate
*Lactobacillus plantarum*	CCFM595	CGMCC9511; Chinese Sichuan pickle isolate
*Lactobacillus plantarum*	CCFM382	CGMCC9734; Chinese traditional leavened isolate
*Lactobacillus plantarum*	CCFM675	CGMCC9662; human feces isolate
*Lactobacillus plantarum*	CCFM734	not available
*Lactobacillus fermentum*	CCFM787	not available
*Lactobacillus fermentum*	CCFM381	Chinese traditional leavened isolate
*Lactobacillus fermentum*	CCFM421	Chinese traditional fermented fish isolate
*Lactobacillus fermentum*	CCFM620	Chinese traditional fermented green beans isolate
*Lactobacillus casei*	CCFM9	Pickle isolate
*Lactobacillus casei*	CCFM30	Cow milk isolate
*Lactobacillus reuteri*	CCFM14	CICC6226; Yoghurt starter strain
*Lactobacillus rhamnosus*	CCFM237	CGMCC7317
*Lactobacillus acidophilus*	CCFM137	human feces isolate
*Streptococcus thermophilus*	CCFM218	Kefir isolate
*Lactobacillus brevis*	CCFM498	Chinese northeast sauerkraut isolate

CCFM refers to Culture Collections of Food Microbiology, Jiangnan University, Wuxi, China.

CGMCC refers to China General Microbiological Culture Collection Center, Beijing, China.

CICC refers to China Center of Industrial Culture Collection, Beijing, China.

**Table 2:** Agonists used for THP1-XBlue™ and HEK-Blue™ reporter cell stimulation assay.

Cell line	Cell density	Specific agonist applied as positive control	Concentration of agonist added to wells
THP1-XBlue^TM^-MD2-CD14	1 × 10^6^ cells/ml	LPS-EK Ultrapure	1 μg/ml
HEK-Blue™ hTLR2	2.8 × 10^5^ cells/ml	FSL-1	50 ng/ml
HEK-Blue™ hTLR3	2.8 × 10^5^ cells/ml	Poly(I:C) LMW	1 μg/ml
HEK-Blue™ hTLR4	1.4 × 10^5^ cells/ml	LPS-EK Ultrapure	1 μg/ml
HEK-Blue^TM^ hTLR5	1.4 × 10^5^ cells/ml	Rec FLA-ST	0.1 μg/ml
HEK-Blue^TM^ hTLR7	2.25 × 10^5^ cells/ml	Imiquimod (R837)	100 μg/ml
HEK-Blue™ hTLR8	2.25 × 10^5^ cells/ml	ssRNA40/LyoVec™	50 μg/ml
HEK-Blue™ hTLR9	4.5 × 10^5^ cells/ml	ODN 2006	2.5 μM
