# Supplementary material for: Identification of TLR2/TLR6 signalling lactic acid bacteria for supporting immune regulation
Source: Sci Rep. 2016 Oct 6;6:34561. doi: 10.1038/srep34561 (PMC5052581; doi:10.1038/srep34561)
Supplement: Supplementary Information [file srep34561-s1.pdf]

Identification of TLR2/TLR6 signalling lactic acid bacteria for supporting immune regulation

Chengcheng Ren[1,2*], Qiuxiang Zhang[2], Bart J. de Haan[1], Hao Zhang[2], Marijke M. Faas[1], and Paul de Vos[1]

[1]Immunoendocrinology, Division of Medical Biology, Department of Pathology and Medical Biology, University of Groningen and University Medical Center Groningen, Hanzeplein 1, 9700 RB Groningen, The Netherlands.

[2]School of Food Science and Technology, Jiangnan University, 1800 Lihu Road, Wuxi 214122, China

[*]Correspondence and requests for materials should be addressed to C.C.R. (email: s.ren@umcg.nl)

Supplementary data

(Legend to Figure S1)

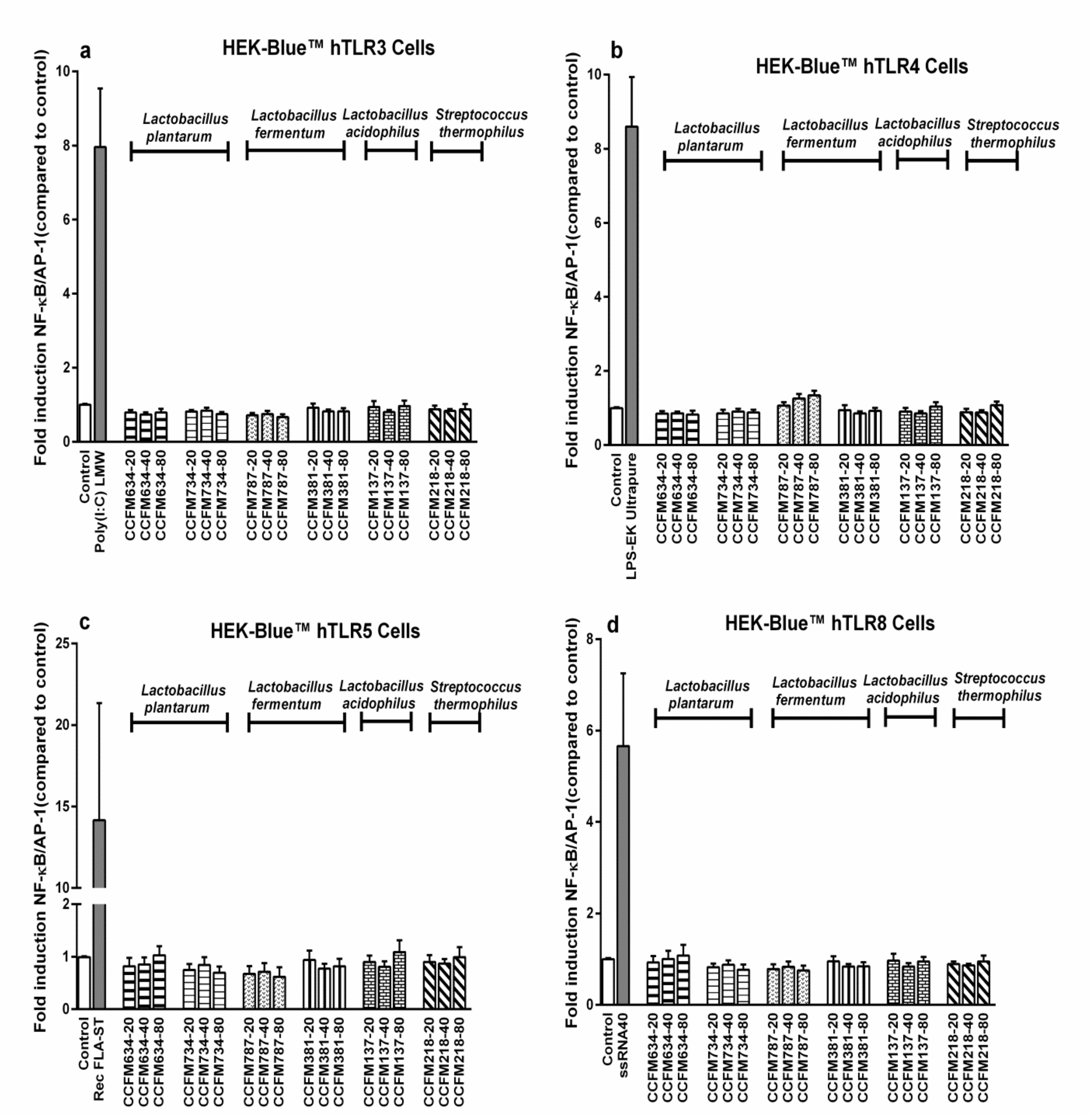

Figure S1. Activation of TLRs pathways by different bacterial strains in HEK-Blue^{TM} reporter cells. A series of HEK-Blue^{TM} TLR reporter cell lines were stimulated with various concentrations of LAB strains. After 24h of co-incubation, SEAP activity in cell culture supernatants was assessed. Respective agonists for TLR served as positive control group. NF-κB/AP-1 activity is presented as percentage of untreated control cells. For each strain, -20, -40 and -80 represent different bacteria/cells ratios respectively. The results shown represent

mean and standard deviation (SD) of three independent experiments. Statistical significance between different treatment groups and untreated control group was measured using one-way ANOVA with Bonferroni multiple comparisons test (*p<0.05; **p<0.01, ***p<0.001).

(Legend to Figure S2)

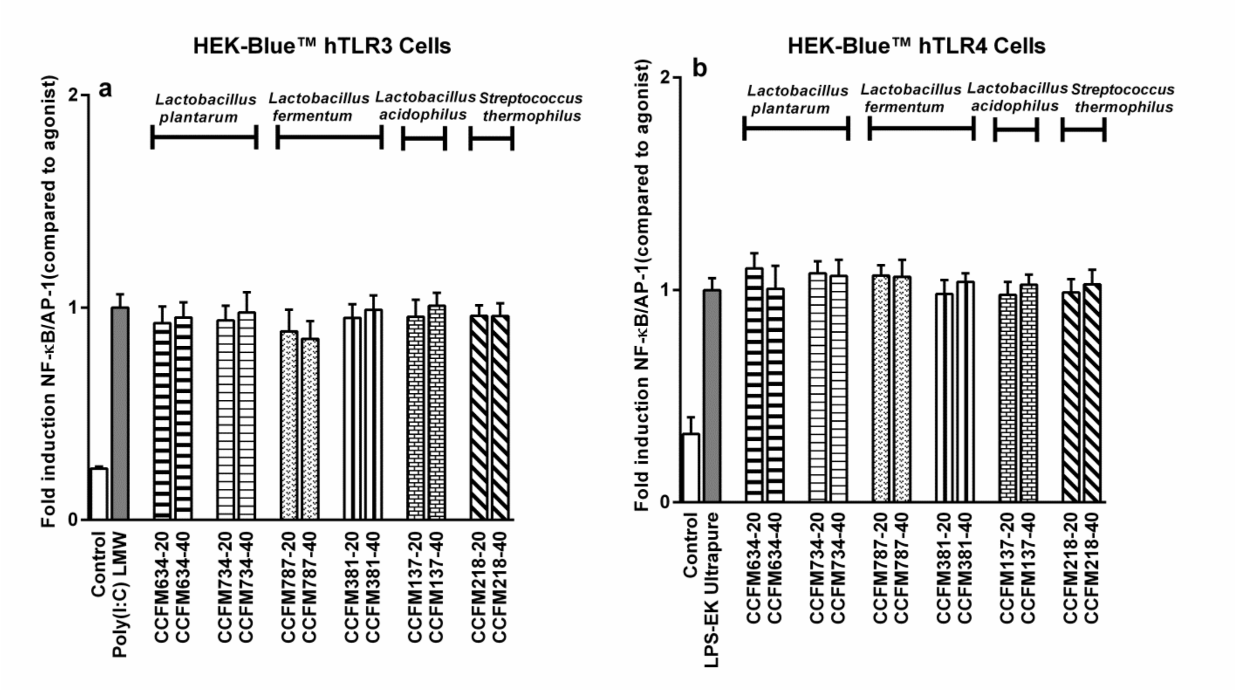

Figure S2. Effect of bacteria on agonists-induced NF-κB/AP-1 activation. HEK-Blue^{TM} TLR reporter cells were stimulated with their agonists and bacteria for 24h. Then SEAP activity in cell culture supernatants was measured. NF-κB/AP-1 activity is presented as percentage of signals induced by respective agonists. For each strain, -20 and -40 represent different bacteria/cells ratios respectively. The results shown represent mean and standard deviation (SD) of three independent experiments. Statistical significance between different bacteria treatment groups and agonists treatment group was measured using one-way ANOVA with Bonferroni multiple comparisons test (*p<0.05; **p<0.01, ***p<0.001).
